# Behavioral trace data in an online learning environment as indicators of learning engagement in university students

**DOI:** 10.3389/fpsyg.2024.1396881

**Published:** 2024-10-23

**Authors:** Marc Winter, Julia Mordel, Julia Mendzheritskaya, Daniel Biedermann, George-Petru Ciordas-Hertel, Carolin Hahnel, Daniel Bengs, Ilka Wolter, Frank Goldhammer, Hendrik Drachsler, Cordula Artelt, Holger Horz

**Affiliations:** ^1^Educational Psychology, Geothe University, Frankfurt, Germany; ^2^Global Affairs Study and Teaching, Statistics Unit, Goethe University, Frankfurt, Germany; ^3^Educational Technologies, Leibniz Institute for Research and Information in Education, Frankfurt, Germany; ^4^Psychological Diagnostic, Ruhr University Bochum, Bochum, Germany; ^5^Technology Based Assessment, Leibniz Institute for Research and Information in Education, Frankfurt, Germany; ^6^Department for Competencies, Personality and Learning Environments, Leibniz Institute for Educational Trajectories, Bamberg, Germany

**Keywords:** learning engagement, trace data, best-subset regression, asynchronous online learning, learning analytics, university student behavior

## Abstract

Learning in asynchronous online settings (AOSs) is challenging for university students. However, the construct of learning engagement (LE) represents a possible lever to identify and reduce challenges while learning online, especially, in AOSs. Learning analytics provides a fruitful framework to analyze students' learning processes and LE via trace data. The study, therefore, addresses the questions of whether LE can be modeled with the sub-dimensions of effort, attention, and content interest and by which trace data, derived from behavior within an AOS, these facets of LE are represented in self-reports. Participants were 764 university students attending an AOS. The results of best-subset regression analysis show that a model combining multiple indicators can account for a proportion of the variance in students' LE (highly significant *R*^2^ between 0.04 and 0.13). The identified set of indicators is stable over time supporting the transferability to similar learning contexts. The results of this study can contribute to both research on learning processes in AOSs in higher education and the application of learning analytics in university teaching (e.g., modeling automated feedback).

## 1 Introduction

Recently reinforced by pandemic circumstances, asynchronous online settings (AOSs) have been on the rise for years and are assumed to continue to blend in the higher education learning landscape (Adedoyin and Soykan, 2020). Even though AOSs are not novel within educational discussions, their implementation entails challenges concerning student motivation, learning activities, and regulation (Fabriz et al., 2021; Hartnett, 2015). Among these, learning engagement (LE) during online learning can largely affect students' learning processes and their outcomes (Nguyen et al., 2021). Students' trace data during online learning were found to be reliable indicators of academic performance and learning-related characteristics (Syal and Nietfeld, 2020). Learning analytics (e.g., Siemens, 2013) stresses that learning context indicators should be identified and selected based on theoretical considerations (Winne, 2020). In this study, indicators are defined based on dynamic activities or context data of learners collected in a virtual learning environment (in a sense of contiguous occurrence referred to as trace data). Trace data have shown the potential to represent LE (e.g., Reinhold et al., 2020), but the relationship between trace data-based indicators and LE, particularly in the context of AOSs, is still unclear. Therefore, this study aims to address this gap by examining the associations between observable learning behavior in an AOS and students' LE to determine how indicators based on trace data can predict students' LE.

Three considerations, consisting of (I) the examination of a representation of LE in the sub-dimensions of effort, attention, and content interest, (II) the representability of the sub-dimensions using 16 potential trace data indicators, and (III) the possibilities of a model-based prediction of LE using linear models, characterize the paper structure.

To test the stability of the aspired models, two lessons are compared that took place at different stages of the same online course. A theoretical excursus on the (importance of) operationalization and measurability of LE is followed by the presentation of the available trace data and an explanation of the modeling method used (best-subset regression).

## 2 Materials and methods

### 2.1 Learning engagement in (online) learning

Learning describes an act of information processing through various levels and units of human memory (see Atkinson and Shiffrin, 1968; Baddeley, 1992). These considerations emphasize learners' active role in cognitive, metacognitive, affective, and motivational processes during learning (see Boekaerts, 1999; Winne, 2001). Given that framework, multi-dimensional constructs seem plausible but also inevitable in regard to describing the learning process, always in a descriptive tradeoff between process components and possible (sub-) outcomes. As LE has predominantly been defined as the observable consequence of learning motivation and participation in learning activities (e.g., Hu and Hui, 2012; Lan and Hew, 2020), the highly complex construct comprises abundant dimensions. Following that understanding of LE's versatility, the construct is frequently divided into behavioral, cognitive, or emotional dimensions which will be captured in this study as effort, attention, and content interest (e.g., Jamet et al., 2020; Lan and Hew, 2018; Deng et al., 2020).

Given those facts, LE is considered a major factor influencing learning outcomes or academic success to the same extent as learning persistence and performance (Kuh et al., 2008). While previous positive effects of LE (e.g., on knowledge retention or processing depth of learning material; e.g., Sugden et al., 2021) retain their relevance beyond traditional learning settings, the role and potential of LE become even more important in learning contexts with time- and location-independent learning activities that rely on high levels of self-control and provide educators with fewer possibilities to moderate learning processes immediately.

In addition, considering that learning is not only an individual but also a social process (e.g., Young and Collin, 2004), all sub-dimensions of LE are relevant in the context of both institutionalized AOSs and non-institutionalized AOSs (e.g., Massive Open Online Courses; MOOCs). Although MOOCs often differ in terms of their learners, for example, because they are primarily independent of university curricula and participants do not identify as students (Watted and Barak, 2018), the role of LE is widely discussed in this domain (e.g., Zhang et al., 2021). With MOOCs and other open educational resources being integrated into institutionalized curricula (e.g., in blended learning; Feitosa de Moura et al., 2021), the lines between MOOCs and institutionalized AOSs are blurred. Not capable of educators' traditional in-class impressions, gained in classical learning settings, a higher value must be placed on learner activities and information that can be derived from their behavior. Accordingly, recent studies explored both sides of the coin, taking into account that learners' active engagement with the learning material in AOSs is a relevant determinant of student learning (cf. Bosch et al., 2021; Koszalka et al., 2021; Lan and Hew, 2020) while underlining the predictability of LE by learner characteristics (Daumiller et al., 2020; Doo and Bonk, 2020; Gillen-O'Neel, 2021). A popular application that uses LE inferences is, for example, in the prediction or determination of drop-out rates (Landis and Reschly, 2013).

#### 2.1.1 Measuring learning engagement

Henrie et al. (2015a) report an imbalance of LE measurement approaches, depicted in 61.1% of studies relying on quantitative self-reports and 34.5% relying on quantitative observational measures (e.g., time-on-task considerations), including technologically sophisticated set-ups that even capture bio-physiological data via sensor.

In addition to other contextual considerations (e.g., experimental set-ups in which data collection occurs and often entails potential validity issues vs. *in-situ* approaches that are contaminated due to the collection procedure itself; Jürgens et al., 2020), measuring LE is likely to succeed through triangulation of multiple approaches (Stier et al., 2020; Ober et al., 2021). Hence, our study builds on the advantages of self-reports (personal insights in cognitive and emotional or motivational processes that precede behavior, and operationalization of abstract concepts) as well as beneficial aspects of observational data (measuring actual behavior that is affected by external circumstances as well as intentions/cognitive and emotional processes). In context of LE measurement, this approach aligns with a recent line of established research (Dixson, 2015; Henrie et al., 2015b; Pardo et al., 2017; Henrie et al., 2018; Tempelaar et al., 2020; Van Halem et al., 2020; Kim et al., 2023).

#### 2.1.2 Operationalization of learning engagement via trace data

In previous learning analytics approaches, the investigation of digital trace data addressed predominantly student academic performance, that is, their learning outcomes (e.g., Caspari-Sadeghi, 2022). Given that students' learning activities precede and affect sustainable and successful learning (Bosch et al., 2021; Ferla et al., 2010), confirmation of the validity of trace data (e.g., Kroehne and Goldhammer, 2018; Goldhammer et al., 2021; Hahnel et al., 2023) regarding LE is essential. Data-driven approaches are often discussed in light of validity or even interpretability and their high dependency on (sample) datasets (e.g., Smith, 2020; Zhou and Gan, 2008) but need also be embedded in theory, that is, for this study briefly illustrated below.

##### 2.1.2.1 Operationalizing behavioral dimensions of LE

Behavioral engagement implies observable (participatory) actions and activities that are linked to favorable circumstances while learning (e.g., in-class/verifiable note-taking, completion of presented videos, number of (forum) postings, and attendance/time on task). With the help of trace data, tendencies of progression (or termination) within courses can be detected, mostly taking into account a wide variety of resources (Deng et al., 2020; Reinhold et al., 2020).

##### 2.1.2.2 Operationalizing cognitive dimensions of LE

Cognitive engagement is often framed in the context of processing theories (e.g., Baddeley, 1992), focusing on the amount and quality of effort invested when interacting with the material. A distinction occurs between routine processing (baseline) and the integration of new knowledge into existing structures (Greene, 2015). Deng et al. (2020) describe such a distinction as “a willingness to exert efforts and go beyond what is required.” In summary, active mental states, tendencies of higher-order thinking, and the ability to be cognizant of the content, meaning, and application of academic tasks (entering a didactical meta-level or at least a state of personal long-term importance) characterize this cognitive dimension (Bowden et al., 2021).

##### 2.1.2.3 Operationalizing affective/emotional dimension of LE

Emotional engagement is by far the most abstract dimension, often described as emotional or affective effort toward learning material. Renninger and Bachrach (2015) capture the essence of this dimension and point out dependencies by proclaiming:

“It is possible to be behaviorally engaged but not interested, whereas it is not possible to have an interest in something without being engaged in some way (e.g., behaviorally or cognitively).”

Since we operationalize this dimension labeled as *content interest*, mentioning its close interconnectedness with cognitive engagement is crucial and has to be interpreted as a coordinated rather than a separated operation (Renninger and Bachrach, 2015). Thus, entering the world of intentions and motivation toward learning, materials, and courses, the concept of self-regulation often accompanies or is used as a proxy for emotional (and sometimes cognitive) engagement since it can be interpreted as effort or strategic or conscious acting put into a matter (Deng et al., 2020; Greene, 2015).

Summing up, possible indicators that can depict the presented thoughts can be found in Table 1. All indicators have been derived from either LE or self-regulated learning literature, were previously used within trace data contexts, and are displayed with the indicator titles of the primary source.

**Table 1:** Presumed LE dimensions, affected by trace data indicators.

**Trace data measurement**	**Previous utilization (indicator title of primary source)**	**Presumed LE dimensions**
More than one quiz attempt	Hershcovits et al., 2020 (repetition rate)	Behavioral
0 = no second quiz-attempt with score < 100%; 1 = second quiz attempt with score < 100% | 100% at first quiz- attempt	Hershcovits et al., 2020 (repetition rate); Aluja-Banet et al., 2019 (persistence indicators)	Behavioral, emotional
L completion within the first 3 days of the processing period	Aluja-Banet et al., 2019 (speed indicators)	Behavioral, emotional
Time difference between the first access/latest submission per assignment	Aluja-Banet et al., 2019 (speed indicators); Li et al., 2020 (study in advance); Baker et al., 2020 (how far in advance students start work on/turn in various assignments)	Cognitive, behavioral
Time difference between first access and the deadline	Li et al., 2020 (study in advance); Baker et al., 2020 (how far in advance students start work on/turn in various assignments)	Cognitive, behavioral
Access to indices of content	Cicchinelli et al., 2018 (Access to indices of content)	Cognitive, emotional
Opening the 'additional content' page (overview)	Aluja-Banet et al., 2019 (choice indicators)	Cognitive, emotional
Visited additional content (hyperlink)	Aluja-Banet et al., 2019 (choice indicators)	Emotional
Number of short breaks	Derived from disengagement literature (e.g., Cocea and Weibelzahl, 2011; O'Brien et al., 2022), considered for a fine granular measurement of time on task	Cognitive, behavioral
Number of long breaks (>15 Min)		Cognitive, behavioral
Access to any resource related to course organization	Cicchinelli et al., 2018 (Access to any resource related to course organization)	Behavioral
Access to pages describing the exercises	Cicchinelli et al., 2018 (Access to pages describing the exercises)	Behavioral
Number of accesses to content pages	Cicchinelli et al., 2018 (Access to content pages)	Behavioral
Access to calendar (deadlines)	Baker et al., 2020 (frequency with which students view resources pertaining to course dates and deadlines)	Cognitive, behavioral
Creation of virtual note	Lin and Tsai, 2012 (Annotations on personal bookmarks)	Cognitive, behavioral
Frequency of clicking the notepad or going back to texts/videos during quiz-attempt	Baker et al., 2020 [preview and review events […] before (preview) or after (review) […] event ([…] quiz, exam, or lecture)]	Cognitive

### 2.2 Research questions

Following the above-mentioned explanations, the study attempts to answer two research questions.

The first research question examines the extent to which trace data (independent variables) can be assigned to respective behavioral, cognitive, or emotional sub-dimensions derived from LE research and its combined potential to explain self-reported LE (dependent variables). Therefore, it is formulated as follows:

**RQ1:** Which student learning behavior (trace data-based indicators) represents students' self-assessed LE?

The second research question deals with the stability of possible model-based predictions. In the sense of measurement repetition, it applies linear models generated using one data subset [lesson 2 (L2); t1] to a comparable dataset [lesson 5 (L5); t2]. The chosen procedure not only makes it possible to check the stability of a model generated at t1 concerning the explained variance over both measurement times but also offers the opportunity to show the extent to which the explained variance of a model optimized at t2 (benchmark) differs from the model generated at t1, providing a framework in which the significance of the context in which learning takes place can be discussed. Therefore, the following formulation adequately summarizes the line of thought.

**RQ2:** Are the interrelations of linear models, which utilize LE indicators, stable over two non-consecutive lessons with different topics?

### 2.3 Methods

#### 2.3.1 Course and procedure

Data were collected in an AOS on teaching with digital media realized via Moodle. The learning management system (LMS) was capable of collecting both trace data and self-reports. It was designed specifically for the objectives of the study. Within the AOS, five consecutive lessons were released on a bi-weekly schedule. All lessons shared a global structure and recurring elements (e.g., texts, videos, and quizzes) but strongly varied in learning activities (notepad, concept map, discussion forum, and self-assessments) and content. To counteract effects caused by the strong variation in learning activities and the associated differences in behavior, we chose lessons that were most comparable and still made it possible to answer the question of temporal stability. L2 (middle of the semester; topic: “Constructivism and digital media”) and L5 (end of the semester; topic: “Individualizing learning processes through the use of media”) are comparable regarding the types of learning material: three texts and three videos followed by a quiz, containing 10 items covering the material. L2 text material had a length of 993, 998, and 961 words, while L5 text material possessed a size of 1,328, 1,493, and 1,578 words. The used videos of L2 were 2:51, 3:14, and 6:15 min long, while L5 videos had lengths of 3:13, 3:35, and 4:54 min. At the end of both lessons, students were asked to fill in a questionnaire regarding their learning behavior during this lesson (see 2.4.1 Measures).

### 2.4 Participants

Participants were 764 teacher students from two German universities. For answering both RQs, subsets of two comparable lessons were used further on (L2: *n* = 608, L5: *n* = 408, Table 2). Both samples derive from the same population, and two different measurement times of the same online course are the subject of the study; therefore, 372 people are included in both datasets, which generate dependencies.

**Table 2:** Descriptive statistics.

		**Gender**				**Age**		**Semester** ^**a**^		
	**N/n** ^b^	**f**	**m**	**d**	**NA**	***M***	***SD***	**1. Sem**	>**1. Sem**	**NA**
Course	764	478	122	2	162	21.73	4.94	668 (86%)	74 (10%)	32 (4%)
L2	607	414	110	2	81	21.86	5.13	466 (77%)	62 (10%)	79 (13%)
L5	406	306	71	2	27	22.31	5.59	334 (82%)	47 (12%)	25 (6%)

Course dataset was used for confirmatory factor analysis (RQ1), L2 subset was used for modeling (RQ2), and L5 subset was used to examine models (RQ2).

a Semester information is displayed to estimate behavioral homogeneity according to previously gained experience while using Moodle as LMS.

b Disparity is due to the fact that only complete datasets (in regard to indicator variables) were used, accompanied by semester drop-out rates.

Since participants actively registered for the AOS, only driven by the pre-released content description and not by an explicit interest in participating in the study, data must be interpreted as results of an *ad-hoc* sample.

The sample consisted of teacher students, who attend different study programs, combining at least two majors (from Natural Sciences, Linguistics, Arts, Sports, Humanities, and modules in Educational Sciences). Given that, the sample is able to incorporate tech-close/-distant study routines (e.g., programming or text-based research-intensive courses vs. laboratory work or gym lessons) that might map LMS-usage variance. Limited by its homogeneity in regard of the student population, the sample yet allowed a non-biased material-wise insight, due to the fact that all participants were interested in the topic of how to teach (the respective content) with digital media. On a voluntary basis, students indicated the following course attendance (multiple selection possible; across all teaching degree programs): 4% Sport, 6% Art, 19% Humanities, 34% Natural Sciences, and 36% Linguistics.

The study was approved by the ethics committee as participation in the AOS was possible regardless of the provision of data. Declarations of consent were obtained from all participants for the data used in the study.

#### 2.4.1 Measures

##### 2.4.1.1 Dependent variables

LE is the dependent variable. It was operationalized as students' effort, attention, and content interest while engaging with the AOS. Data were collected from self-reports at the end of every lesson. These dimensions were measured via adapted versions of the MOOC Learner Engagement and Motivation Scale (MEM; Lan and Hew, 2018) and the MOOC Engagement Scale (MES; Deng et al., 2020), using only the subscales for emotional and behavioral engagement. Adaptions have been made in linguistic terms, (I) translating the scales into German, (II) referring to “lessons”, instead of “MOOCs” and in methodological terms by (III) using a 4-point Likert scale to strengthen response tendencies (instead of the original 5-/6-point Likert). Effort, attention, and content interest were remodeled in the sense of factors within the chosen item pool (effort: MEM, subscale behavioral engagement, items 1 and 2; attention: MEM, subscale behavioral engagement, items 4 and 5; content interest MEM subscale emotional behavior, items 4 and 5; MES subscale emotional behavior, item 9; Table 3). A confirmatory factor analysis supported the assumed factor structure [χ^2^_(11)_ = 105.94; *p* < 0.001; CFI = 0.992; RMSEA = 0.051; SRMR = 0.024].

**Table 3:** Reliability and descriptive statistics of used instruments and remodeled LE dimensions.

	***M***	***SD***	**Min**	**Max**	**Cronbach's α**	**McDonald's ω**
MEM; Lan and Hew (2018)	3.13	0.50	1	4	0.81	Total: 0.91
MES; Deng et al. (2020)	2.70	0.56	1	4	0.62	Total: 0.75
Remodeled: effort	3.47	0.62	1	4	0.84	Factor: 0.82 total: 0.92
Remodeled: attention	3.30	0.62	1	4	0.84	Factor: 0.88; total: 0.92
Remodeled: content interest	3.04	0.65	1	4	0.84	Factor: 0.85; total: 0.92

##### 2.4.1.2 Independent variables

Independent variables were derived from the behavioral trace data. Indicators were created as dichotomous (e.g., successful completion of lesson within the first 3 days) or interval-scaled variables (e.g., time on task). The indicators refer to trace data collected while students interacted with organizational (e.g., overview page) and learning material pages (i.e., instructions, texts/videos, quiz, and concept map activities).

For answering the postulated RQs, we opted for an approach that allowed us to design the learning environment taking into account both learning analytics considerations and the ideas of instructional design (see FoLA2 by Schmitz et al., 2022), as well as literature-based classification approaches, such as “*time sum*” (e.g., Baker et al., 2020), “*timing of an action*” (Coffrin et al., 2014; Wang et al., 2019), “*action occurred (dichotomous)*” (e.g., Cicchinelli et al., 2018), “*action count*” (e.g., Baker et al., 2020; Cicchinelli et al., 2018; Jovanović et al., 2019), and “*time difference”* (e.g., Li et al., 2020) (Table 4).

**Table 4:** Operationalizing behavioral, cognitive, and affective/emotional dimensions of LE.

**Indicator**	**Measurement**	**Min**	**Max**	**M**	**SD**
10012	More than one quiz attempt^c^	0	1	0.81	-
10061	L completion within the first 3 days of the processing period^b^^,^^c^	0	1	0.13	-
10141	Opening the 'additional content' page^c^	0	1	0.28	-
10152	Visited additional content (Hyperlink)^c^	0	1	0.03	-
10551	Creation of virtual note^c^	0	1	0.08	-
10591	0 = no second quiz-attempt with score < 100%; 1 = second quiz attempt with score < 100% | 100% at first quiz- attempt^c^	0	1	0.80	-
1071	Number of short breaks (> 80 s, < 15 min)^d^	0	87	13.08	11.83
1072	Number of long breaks (> 15 min)^d^	0	28	2.12	3.12
2001	Access to any resource related to course organization^c^	0	98	7.38	8.56
2002	Access to indices of content^c^	0	79	10.91	9.07
2003	Number of accesses to content pages^d^	0	130	12.32	12.23
2004	Access to pages describing the exercises^d^	0	11	0.51	1.04
2011	Access to calendar (deadlines)^d^	0	4	0.22	0.60
2012	Time difference between the first access/latest submission per assignment^a^	323	1,140,046	1,81,916	2,81,767
2016	Time difference between first access and the deadline^e^	6,859	1,212,978	7,00,923	3,82,749
2022	Frequency of clicking the notepad or going back to texts/videos during quiz-attempt^d^	0	172	5.53	16.02

Presented descriptive statistics refer to L2 data.

a time sum (seconds),

b timing of an action (dichotomous; timing defined as a condition),

c action occurred (dichotomous),

d action count (metric),

e time difference (seconds).

##### 2.4.1.3 Data curation

Dealing with missing data on not one but two data streams requires strict curation logics. Self-report data have been selected preferably due to its function to represent dependent variables. In a first step, students who did not meet the passing requirements of the course were removed. Those requirements were defined as active participation, including a quiz at the end of every lesson (non-graded, unlimited repeatable) and completing a self-report. Regarding the self-report data, no imputation took place but complete case datasets were used, resulting into 764 users, either participating in L2, L5, or both. Within the trace data stream, one indicator has been deleted completely since the computational logics failed (ca. 80% NAs). No further outlier detection was undertaken (e.g., with the help of confidence intervals), but the raw data were used. NA curation did not take place since the later modelation approach only incorporated cases with the recommended variables.

##### 2.4.1.4 Data analysis

To answer **RQ1**, we first computed bivariate correlations between the indicators and the LE dimensions of effort, attention, and content interest using the statistics software R (version 4.3.0; *Hmisc* package; Harrell, 2023). Afterward, to maximize the explained variation, we identified regression models to predict each LE dimension derived from the trace data-based indicators. Doing so, we used a best-subset regression approach (cf. King, 2003), opting for this method for the following reasons: While stepwise selection methods mostly report only one single model, the logic of best-subset regression analysis is to report multiple models, often statistically indistinguishable in a set of alternative models, yet varying the independent variables usage (cf. Hastie et al., 2020). Doing so, a best-subset approach is theoretically capable to compute models without a rigorous need for thresholds/penalties (with the drawback of computational power, cf. Furnival and Wilson, 1974), which is common in classic modelations, frequently imposed by the Akaike information criterion (Reiss et al., 2012). Consequently, best-subset regression analysis allows researchers to interpret sets of models by non-preselected coefficients more unbiased. Following these methodological considerations, we computed nine models per LE dimension, incorporating up to nine predictors (*leaps* package v3.1; Lumley and Miller, 2020; the number of nine predictors is due to the computational limitations of the package and not theory-driven). Afterward, validation of models followed a R^2^ orientation to identify global maximal values (cf. Akinwande et al., 2015). In the last step, the plausibility of the variables was taken into account regarding their generalizable fit/contextual robustness (e.g., the possibility of replication with the perspective on measurement, i.e., time on tasks that occur in both lessons). It should be emphasized that the R^2^ and adjusted R^2^ values remain unaffected during the described plausibility check, still aspiring to select the models with the highest R^2^. In regard of plausibility as selection criterion, models with the highest performing R^2^ were always considered most robust, so no further semantical or logical decisions needed to me made.

The previously described data proceeding steps made it possible to (a) get a detailed overview of the impact of the addition or replacement of aspects of student learning behavior (indicators) that reflect respective associated students' self-assessed LE while (b) semantically identifying the most robust model, incorporating the least context-sensitive indicators. To answer **RQ2**, the final regression models, which were identified for L2, were transferred and tested on L5 data (*MASS* package; Venables and Ripley, 2022). Since we only used complete cases in all our procedures, we checked variables that mostly revealed missing values beforehand for not unnecessarily downsize the potential variables for regression models (Table 2).

## 3 Results

### 3.1 RQ1: individual indicators representing learning engagement

The results for both lessons L2 and L5 showed weak bivariate correlations of individual indicators with the LE dimensions of effort, attention, and content interest (Tables 5, 6). While 26 correlations of 16 unique indicators were significant (*p* < 0.05), the correlations of only three unique indicators were relevant in both lessons, respectively (r ≥ 0.1 (rounded); 10012, 1071, 2022).

**Table 5:** Bivariate correlations (Spearman's) of ordinal-scaled L2 and L5 indicators with LE dimensions.

	**Effort**		**Attention**		**Content interest**	
Indicator	L2	L5	L2	L5	L2	L5
1071	0.14^***^	0.13^*^	0.14^***^	0.05	0.16^****^	0.11^*^
1072	0.09^*^	0.09	0.09^*^	−0.01	0.06	0.13^*^
2001	0.09^*^	−0.07	0.07	−0.04	0.08	0.1^*^
2002	0.07	−0.05	0.07	−0.09	0.08	0.04
2003	−0.04	−0.01	−0.05	−0.06	0.01	0.00
2004	0.03	0.03	0.05	−0.03	−0.02	0.03
2011	−0.01	0.08	−0.03	0.12^*^	0.02	0.2^****^
2012	0.05	0.00	0.02	−0.05	0.03	0.13^*^
2016	0.12^**^	0.13^*^	0.07	0.05	0.01	0.08
2022	−0.11^**^	−0.16^**^	−0.16^****^	−0.11^*^	−0.06	−0.09

* p ≤ 0.05,

** p ≤ 0.01,

*** p ≤ 0.001, and

**** p ≤ 0.0001.

**Table 6:** Bivariate correlations (Pearson's; point biserial) of dichotomous L2 and L5 indicators with LE dimensions.

	**Effort**		**Attention**		**Content interest**	
Indicator	L2	L5	L2	L5	L2	L5
10012	0.11^**^	0.08	0.09^*^	0.07	0.08	0.08
10061	0.03	0.14^**^	0.04	0.11^*^	0.01	−0.08
10141	0.03	0.01	−0.01	0.03	0.07	0.05
10152	0.02	0.08	0.01	0.08	−0.04	0.05
10551	0.03	0.11^*^	0.04	0.01	0.04	0.10^*^
10591	0.11^**^	0.08	0.09	0.07	0.08	0.08

* p ≤ 0.05 and

** p ≤ 0.01.

Taking into account the used indicator pool and its mostly weak correlation toward the respective LE dimensions, it has to be stated that there are existing weak-to-moderate correlations, mostly highly significant, which could justify the existence of suppressor variables affecting the designed models (Table 7).

**Table 7:** Bivariate correlations (Spearman's) of ordinal-scaled indicators within L2 data.

	**1071**	**1072**	**2001**	**2002**	**2003**	**2004**	**2011**	**2012**	**2016**
1072	0.46^****^								
2001	0.25^****^	0.39^****^							
2002	0.29^****^	0.38^****^	0.73^****^						
2003	0.34^****^	0.30^****^	0.32^****^	0.35^****^					
2004	0.20^****^	0.19^****^	0.31^****^	0.40^****^	0.22^****^				
2011	0.03	0.14^***^	0.21^****^	0.15^***^	0.04	0.02			
2012	0.39^****^	0.51^****^	0.48^****^	0.44^****^	0.31^****^	0.16^****^	0.10^*^		
2016	0.08^*^	0.17^****^	0.14^***^	0.09^*^	0.06	0.02	−0.03	0.36^****^	
2022	0.04	−0.02	0.10^*^	0.06	0.59^****^	0.04	0.04	0.04	0.02

* p ≤ 0.05,

*** p ≤ 0.001, and

**** p ≤ 0.0001.

The regression models for each LE dimension identified from best-subset regressions for the L2 data are shown in Table 8. The models were composed of nine indicators from a pool of 16 unique indicators.

**Table 8:** Standardized beta weights of best subset regression models, modeled with L2 data, examined on L5 data.

		**Learning engagement**		
**Indicator**	**Measurement**	**Effort**	**Attention**	**Content interest**
	Intercept	3.478^***^ (0.02)	3.237^***^ (0.02)	3.037^***^ (0.03)
10012	More than one quiz attempt^c^	0.052^*^ (0.02)	-	-
10061	L completion within the first 3 days of the processing period^b,c^	−0.042 (0.03)	-	-
10141	Opening the “additional content” page^c^	-	-	0.042 (0.03)
10152	Visited additional content (Hyperlink)^c^	-	-	−0.037 (0.03)
10551	Creation of virtual note^c^	0.028 (0.03)	0.035 (0.02)	0.046 (0.03)
10591	0 = no second quiz-attempt with score < 100%; 1 = second quiz attempt with score < 100% | 100% at first quiz- attempt ^c^	-	0.042 (0.02)	0.04 (0.03)
1071	Number of short breaks (> 80 s, < 15 min)^d^	0.092 (0.03)	0.095^***^ (0.03)	0.074^**^ (0.03)
1072	Number of long breaks (> 15 min)^d^	-	0.021 (0.03)	-
2001	Access to any resource related to course organization^c^	0.041 (0.03)	-	-
2002	Access to indices of content^c^	-	-	0.045 (0.03)
2003	Number of accesses to content pages^d^	−0.0451 (0.03)	−0.102^**^ (0.04)	-
2004	Access to pages describing the exercises^d^	−0.024 (0.03)	-	−0.081^**^ (0.03)
2011	Access to calendar (deadlines)^d^	-	−0.023 (0.02)	−0.019 (0.03)
2012	Time difference between the first access/latest submission per assignment^a^	−0.073^*^ (0.03)	−0.039 (0.03)	−0.025 (0.03)
2016	Time difference between first access and the deadline^e^	0.115^***^ (0.03)	0.055^*^ (0.03)	-
2022	Frequency of clicking the notepad or going back to texts/videos during quiz-attempt^d^	-	0.036 (0.04)	-

^*^p ≤ 0.05, ^**^p ≤ 0.01, and ^***^p ≤ 0.001.

### 3.2 RQ2: stability over two non-consecutive lessons/measurement repetition

To examine the stability of the identified models, we applied them on L5 data. Table 9 shows similar R^2^ values for the L2.model on both, L2 as well as L5, datasets. Surprisingly, the R^2^ values on the L5 data are slightly higher. Optimizing the model fit on L5 data by repeating all previous analysis procedures (computing a L5.model on L5 data), displayed in an L5.model R^2^ benchmark, puts the potential of the L2.models in perspective to explain 53–60% of the variance of the optimized model but also highlights further amplification effects evoked by a contextual indicator fit.

**Table 9:** R^2^ values for models based on various subsets.

	**L2.model R** ^**2**^		**L5.model R^2^^a^**
**Dimension/reference dataset**	**L2**	**L5**	**L5**
Effort	0.06^****^	0.07^****^	0.13^****^
Attention	0.05^****^	0.04^*^	0.08^****^
Content Interest	0.04^***^	0.06^**^	0.10^****^

Significance of models that results in presented R^2^ values.

* p ≤ 0.05,

** p ≤ 0.01,

*** p ≤ 0.001, and

**** p ≤ 0.0001.

a achieved benchmark R^2^ from a model explicitly computed with L5 data.

Bootstrapping results (5,000 repetitions) confirm the robustness of the models (effort R^2^: L2 model: 95%CI [0.02,0.08], M:0.07, SD:0.02; L2 model applied on L5 data: 95%CI [0.02,0.10], M:0.09, SD:0.03; attention R^2^: 95%CI [0.02,0.07], M:0.07, SD:0.02; L2 model applied on L5 data: 95%CI [0.01,0.06], M:0.06, SD:0.02; content interest R^2^: 95%CI [0.01,0.05], M:0.05, SD:0.02; L2 model applied on L5 data: 95%CI [0.02,0.10], M:0.08, SD:0.02). Regarding the given data structure, it should be critically noted that linear dependencies exist within the models, which make it necessary to take into account the earlier presented bivariate correlations and the nature of some indicators. Calculation of the variance inflation factors for all models, however, shows that this circumstance has hardly any influence on the presented process (VIFs: effort: min 1.02, max 1.88; attention: min 1.02, max 2.19; content Interest: min 1.02, max 1.46).

## 4 Discussion

This study demonstrates one of the attempts to apply findings from the field of LE research, especially the suppositions that LE dimensions of effort, attention, and content interest vary in their complexity concerning definability, detectability, and occurrence in research (Henrie et al., 2015a,b), for exploring of real-life trace data during learning. For this purpose, a number of indicators were derived from the existing literature and linked to the respective LE dimension (Table 1). While some of the deployed indicators seemed not plausible at first sight, thinking about the implications that need to be met to get specific results, it is still possible to draw conclusions after sharpening their function within models over time, even if it also implies the existence of strong correlations and opens space for suppressor variables (e.g., initially irritated as to why many breaks served to explain effort, the answer lies in the fact that the probability of making those in the first place but also their number increase with the time invested in learning resources; Akinwande et al., 2015). Regarding the explanation of self-reported LE, the study was able to not only prove an insufficient orientation on bivariate correlations but also underline the importance of combining sets of indicators, incorporated in linear models to clarify variance in the dimensions of effort, attention, and content interest. Furthermore, this thought also implies a plausible functioning differentiation in behavioral, cognitive, and emotional characteristics, derived from a stream of action during the learning process (Caspari-Sadeghi, 2022). Even though the respective R^2^ of content interest outperforms attention, the dimension was the hardest to operationalize, mainly enabled by the highest incorporation of dichotomous indicators, which in turn speaks for a vestigial model. After considering learning as sequences of actions, not only representing behavioral but also cognitive and emotional information, observed small-to-moderate explained variation effects (Cohen, 1988) are less surprising, given the complexity inherent in the learning process *per se*. More noteworthy and unexpected is the significance offered by the presented models, particularly in maintaining their validity across repeated measurements and when dealing with a common-sized dataset.

### 4.1 Limitations and further research

The question of generalizability, the given course structure, and dependencies within the study design mark the major hurdles of the study.

In addition to all thoughts invested into the presentation of why the given sample is capable to come with an acceptable amount of behavioral variance (age, semester, and study routines derived from various study majors), the study's external validity needs to be tested. A similar problem needs to be faced in the context of the used questionnaires. Although the factor structure within self-reported LE was examined and found to be conclusive, it remains unclear what specific proportion of self-report is predicted. Future work should address the relationship between LE and performance, as well as continue to model LE as a (robust) latent construct (Wong and Liem, 2022).

Despite the AOS comprising five lessons, only two lessons, nearly identical in their instructional design and structure, were comparable, due to the context-specific characteristics of indicators. While normalization and standardization of time-on-task indicators on video or text might still be possible (e.g., Crossley et al., 2023), other ordinal data such as action counts or time differences are heavily influenced by learning design elements (e.g., tasks that come along with vast behavioral changes such as group work/forums, concept maps, or deadlines within lessons; Ahmad et al., 2022). Next, the role of (inter-)personal characteristics, offline activities in general and especially regarding the intensity of peer interactions during the course, was not investigated in the study. Even though the original study design was capable to address such considerations, we opted for a narrower, yet in the sense of the assessment—better communicable, and more comprehensible way but underline the lack of a by far holistic perspective that needs to be met by further research. Therefore, further studies are needed to investigate more holistic models of further generalizable indicators that can be applied to various contexts.

Finally, the study setup itself is susceptible to dependencies, starting with the fact that the dataset is the product of a university course and therefore 92% of L2 students are part of the L5 dataset, which contributes significantly to answering the question of stability. While this situation could be resolved through cohort comparisons, another issue arises through students' familiarization with the LMS and the question of how to define sort of baseline behaviors. Regarding indicator selection, not only context fit but also habituation effects (e.g., focused vs. exploratory navigation/more deliberate or strategic behavior per se, but also customization status toward the LMS in general; power/expert vs. novice users) need to be discussed with the perspective of its impact on changing baseline attributes in more detail in future research.

Beyond conceptual considerations, this argumentation line must be supplemented at the level of the existing data structure as follows. The existence of moderating, mediating, or suppressor variables as well as collinearity is nurtured throughout the research project due to three factors: (a) by indicator inherent correlations, (b) by the design of the used behavioral indicators *per se*, and (c) by the applied best-subset regression method. All factors are intertwined, which makes a holistic representation of influencing factors almost impossible. To report the mechanisms of action as transparently as possible anyway, examples are given below.

Inherent correlations can be shown, for example, by the situation that the number of breaks taken helps to explain the LE dimension effort, in which the probability of the number of breaks increases with the time invested in processing learning resources. However, the estimated fundamental force at work here lies in the time-on-task logic. The significance of the design of behavioral indicators, on the other hand, can be outlined by focusing on such two indicators as “opening the additional content page” and “visited additional content”, as one indicator is a prerequisite for initiating the other one. Finally, the best-subset regression approach used addresses precisely the mechanisms described here by declaring its aim to include collinear independent variables in model proposals instead of ignoring them due to penalties. However, this increases the probability of the effect of unnoticed suppressor variables, which in doubt reduces effects of not only one but also several independent variables of the proposed models. To put this in perspective, we are aware about the capabilities of machine learning techniques that would probably be able to be more precise concerning modeling but want to point out that the final selection would no longer happen in a sense of deductive, prior assumption-made manner but more on performance. This in turn would contradict the previously outlined balance of interpretability and performance. Knowing about the possibilities of more flexible non-linear modeling approaches, we made a very conscious decision in favor of the chosen path, deploying variables that reflect all three dimensions of LE, not interested in eventually downsizing dimensions by ignoring conceptual interpretability (Luan and Tsai, 2021; Hellas et al., 2018; Khosravi et al., 2022).

Overall, we call for a future research orientation that contributes to assessment considerations, indicator and learning design, firmly anchored in theory.

## 5 Conclusion

This study aimed to investigate the potential to map the complex concept of LE in trace data and, therefore, examined the interrelation between different dimensions of self-reported LE and indicator-based trace data. Best-subset models were used to predict weak-to-moderate proportions (Cohen, 1988) of variance of self-reported statements related to LE. These proportions were confirmed from a repeated measurement perspective, tested on two non-consecutive lessons. The results support the orientation toward a combination of multiple indicators to represent complex constructs such as LE and build a gap between psychometrics and learning analytics.

This representation of complex constructs in trace data can be helpful not only to provide a theoretical background for the interpretation of logged student behavior during learning but also to enable a helpful framework for feedback dashboards that are presented to learners. These dashboards often contain a wide range of information on learner behavior, while the transfer to and the interpretation in regard of successful learning activities remains unclear possibly preventing positive effects of the provided feedback. Well-designed and easily interpretable indicator-based information not only serves as a source for individualized feedback *per se* but also can respond to personal development trajectories while keeping their objectivity stable over time. To establish such a feedback dashboard, a learning analytics design that follows an educational support function and not given technical solutions is crucial. The approach of predicting complex constructs from a set of indicators will contribute to further development of feedback dashboards in learning analytics that focus on adequate communication and concrete suggestions for learning improvement.

## Data Availability

The raw data supporting the conclusions of this article will be made available by the authors, without undue reservation.
